# A rare case report of hyaline-vascular type Castleman disease in the presacral region

**DOI:** 10.3389/fonc.2024.1434542

**Published:** 2024-08-26

**Authors:** Long Chang, Shuang Wang, Jiannan Li, Zeyun Zhao, Min Wang

**Affiliations:** Department of Colorectal and Anal Surgery, Second Affiliated Hospital of Jilin University, Changchun, Jilin, China

**Keywords:** Castleman disease hyaline-vascular variant, presacral mass, unicentric Castleman disease (UCD), surgery, MRI, CT, pathological examination diagnosis

## Abstract

Castleman disease (CD), also known as giant lymph node hyperplasia or angiofollicular lymph node hyperplasia, is a rare and indeterminate group of chronic lymphoproliferative disorders. CD is highly heterogeneous, classified into unicentric Castleman disease (UCD) and multicentric Castleman disease (MCD) based on lesion distribution, and further categorized into three pathological types: hyaline vascular type (HV), plasma cell type (PC), and mixed type (Mix). This paper describes a rare case of solitary mediastinal Castleman disease with transparent vessels in the anterior sacrum, presenting as the HV type. Surgical excision of the mass was performed following coccygectomy for treatment. The patient recovered well postoperatively. During a 6-month follow-up period, there were no signs of recurrence, and the patient’s quality of life significantly improved.

## Introduction

Castleman disease (CD), also known as giant lymph node hyperplasia or angiofollicular lymph node hyperplasia, is a rare and indeterminate group of chronic lymphoproliferative disorders (1). CD, first reported by Benjamin Castleman in 1956, mostly comprises benign conditions and is often misdiagnosed clinically. CD can be classified into unicentric Castleman disease (UCD) and multicentric Castleman disease (MCD) based on the distribution of lesions (1–3). According to pathological classification, CD can be divided into three types: hyaline -vascular type (HV), plasma cell type (PC), and mixed type (Mix). HV type is characterized by lymph nodes containing hyaline vascular sinuses rich in blood vessels, while PC type is mainly characterized by plasma cell proliferation within lymphoid follicles, and Mix type includes both pathological features (4). The precise etiology and pathogenic mechanisms of CD are not yet fully understood. However, the cytokine interleukin-6 (IL-6) is currently considered to be closely associated with the disorder. Given the significant heterogeneity among the subtypes of CD, the mechanisms specific to each category warrant separate discussion. Severe complications can occur in the course of hyaline vascular UCD, such as paraneoplastic pemphigus (PNP), obliterative bronchiolitis, and myasthenia gravis (MG) (5). The occurrence of these complications is considered a potential turning point in the disease’s progression, generally indicating a poorer prognosis for UCD patients. Notably, PNP has been identified as an independent factor associated with increased mortality in UCD, with several studies documenting this association (5–7). Therefore, early detection and complete surgical resection are significant for the effective treatment of UCD (8, 9). Currently, for UCD, complete surgical resection of the lesion is the preferred primary treatment modality. Even in cases where complete resection is not feasible, partial excision can still reduce the recurrence rate. In the presence of contraindications to surgery, consideration may also be given to monotherapy or combination therapy involving radiation and chemotherapy. Treatment plans should be individualized based on the patient’s specific circumstances and the characteristics of the lesion to achieve optimal therapeutic outcomes. In the context of UCD, the HV subtype accounts for nearly 90% of cases, with the mediastinum and cervical lymph nodes being the most common sites affected by HV-type CD among the three pathological subtypes of UCD (10, 11) (12). Over 70% of UCD cases occur in the chest, with the vast majority located in the mediastinum, approximately 15% occur in the neck, and there are also occasional cases occurring in the abdomen and pelvis, mainly affecting lymphoid tissue. Extralymphatic sites affected include the lungs, throat, salivary glands, pancreas, meninges, and muscles (13). Studies have reported that only about 7% of UCDs are located in the retroperitoneal area, with lesions in the abdomen and retroperitoneum being more common in MCD (14). Therefore, UCD occurring in the presacral region is very rare. Reports occasionally describe involvement of lymph nodes in other sites (such as the abdomen, pelvis, and axilla) as well (3, 8, 9) (13). Only about 7% of cases are reported to be located in the retroperitoneal area. The case presented in this article belongs to the HV subtype of UCD, with the lesion located anterior to the sacrum, which is a rare occurrence. This case report presents an in-depth analysis of a patient with CD, specifically highlighting the rarity of its occurrence in the pre-sacral space. This is the first documented instance of UCD manifesting in this anatomical location. Through meticulous clinical evaluation, radiological imaging, and histopathological scrutiny, we successfully diagnosed and characterized this unique presentation of UCD. Complete resection of the lesion was performed after coccygectomy, and the patient showed satisfactory recovery during the 6-month postoperative follow-up.

## Case report

**Figure 1:**
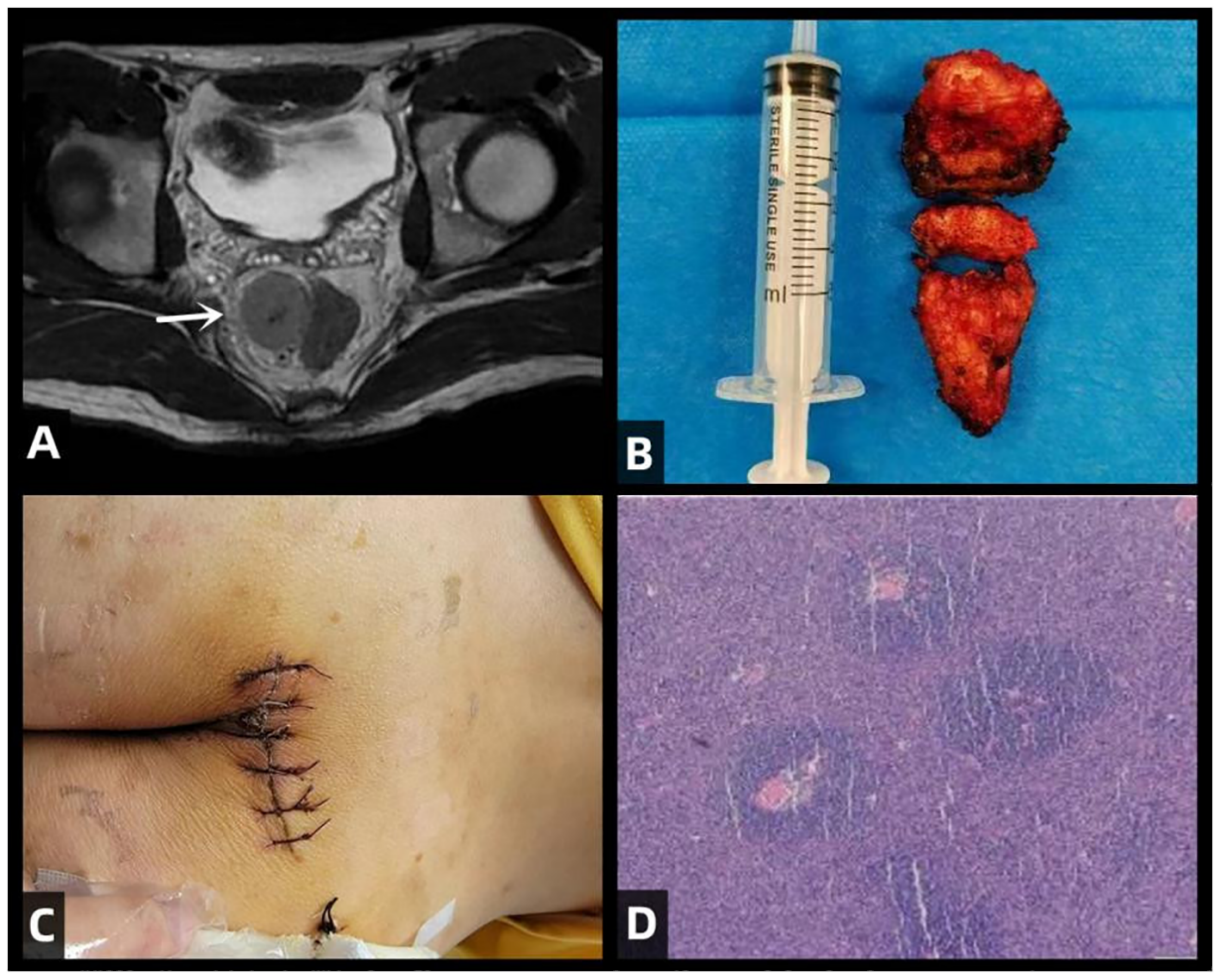
illustrates the clinical features of this patient with CD, including preoperative MRI scans, the surgical excision of the tumor specimen, postoperative incision site, and H&E (hematoxylin and eosin) staining of the excised specimen. **(A)** Rectal mass is observed in the lower right section (white arrow), appearing as a clumped elongated T1 and T2 signal shadow, measuring approximately 31mm × 28mm × 32mm. **(B)** Excised mass from the sacrum: A piece of irregular gray-brown tissue, measuring 4cm in diameter, with a tough whitish texture at the incision site. **(C)** Postoperative incision. **(D)** Histopathological examination of the surgical specimen with H&E staining. The lymph nodes exhibit lymphoid follicular hyperplasia with increased vascularity in the follicular centers. The blood vessels show hyaline changes in their walls, and the germinal centers are reduced in size. Within these centers, follicular dendritic cells with vesicular nuclei display a “burned-out” appearance. The mantle zone small lymphocytes are arranged in concentric circles, resembling “onion skin” patterns.

This study was approved by the Ethics Committee of Jilin University Second Hospital. The patient had no significant complaints prior to admission. The main reason for seeking medical attention was the findings of an enhanced pelvic-prostate magnetic resonance imaging (MRI) scan at a local hospital, which indicated an abnormal signal on the right side of the anterior sacrum, with a high possibility of giant lymph node hyperplasia. Therefore, the patient came to our department for surgical treatment. Preoperative examinations included pelvic MRI scans and colonoscopy. The pelvic MRI showed a mass with a lump-shaped long T1 and long T2 signal in the lower right section of the rectum, measuring approximately 31mm×28mm×32mm. Diffusion-weighted imaging (DWI) showed a high signal, and the apparent diffusion coefficient (ADC) showed a low signal, with unclear boundaries between the lesion and the adjacent lower rectum (**Figure 1A**). Colonoscopy revealed that the tumor did not invade the rectal lumen. Surgery was performed with a midline incision at the sacrococcygeal level, approximately 6cm in length. The skin, subcutaneous tissue, superficial fascia, and deep fascia were sequentially dissected. The coccyx tip and the fifth sacral vertebra (S5) were freed and resected, and the section was coated with bone wax. Eventually, a solid mass was found within the rectal mesentery, extending anteriorly to the sacrum with an intact capsule. The mass was completely excised along the avascular plane, and bleeding points on the wound were electrocoagulated (**Figure 1B**). Digital rectal examination did not reveal any rectal injury or rupture. The residual cavity was irrigated with saline solution, and a curved drainage tube was placed inside the cavity, with one end punctured through the skin on the inner side of the right buttock and sutured in place. The incision was closed in layers (**Figure 1C**), and the surgery was completed.

**Figure 2:**
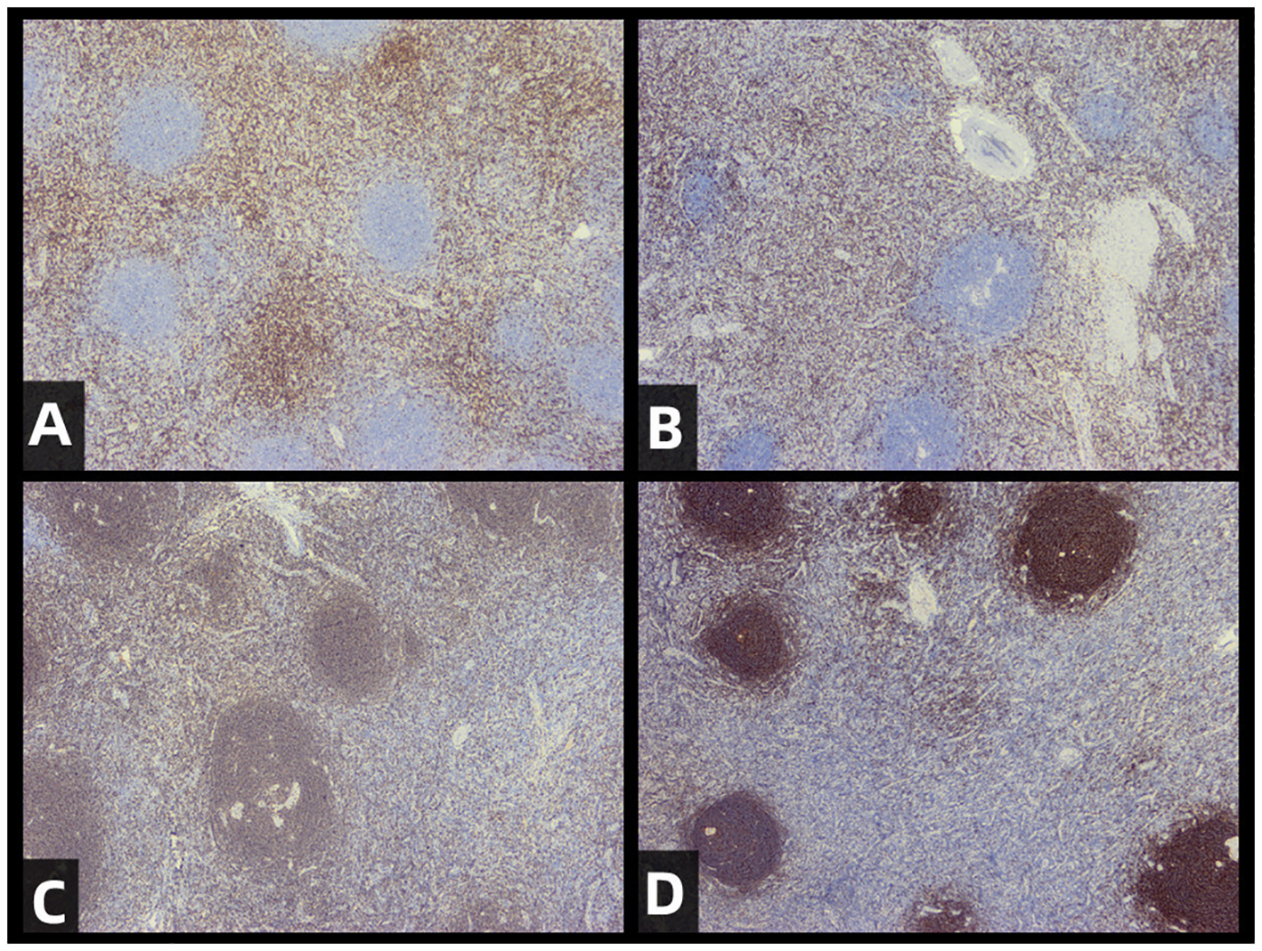
Immunohistochemical staining of resected tumors. The staining results for CD3 **(A)**, CD5 **(B)**, CD20 **(C)**, and CD21 **(D)** are provided to support the diagnosis of CD (castleman disease).The results are as follows: CD5 (positive in the interfollicular area), CD3 (positive in the interfollicular area), CD20 (partially positive), CD21 (positive in follicular dendritic cells).

The pathological diagnosis, combined with immunohistochemical staining results and morphological features, supports the diagnosis of the hyaline vascular type of CD for the excised pelvic mass. (**Figure 1D**) Immunohistochemical staining results showed: CD3 (follicular interstitial area +) (**Figure 2A**), CD5 (follicular interstitial area +) (**Figure 2B**), CyclinD1 (-), CD20 (partially +) (**Figure 2C**), PAX-5 (partially +), CD21(FDC +) (**Figure 2D**), Bcl-2 (germinal center -), Bcl-6 (germinal center -), CD10 (germinal center -), Ki67 (positive rate 5%), HHV8 (-), CD30 (occasional +), EBER (-), CD38 (plasma cells +).

The patient’s pathology staining for HHV is negative, and immunological indicators show that the patient is not infected with HIV. After pathological diagnosis, the patient underwent additional physical examinations, which did not reveal any definite symptoms related to CD, such as fever, night sweats, myasthenia gravis, or rash. The patient did not receive additional chemotherapy after surgery. Follow-up visits were conducted every month postoperatively. The patient’s main complaint postoperatively was discomfort in the lower abdomen influenced by positional factors, primarily manifesting as discomfort when lying supine or reclining, with symptoms alleviating upon changing position. Presently, six months postoperatively, the discomfort has significantly decreased, with no other complaints.

## Discussion

CD’s different subtypes may share common histopathological features. However, due to their distinct sites of occurrence and clinical symptoms, treatment approaches generally vary, leading to potentially different treatment outcomes (15). Due to the low clinical incidence of CD, its detection, differentiation, and diagnosis remain challenging in medical practice (16). CT and MRI imaging techniques alone cannot definitively diagnose Castleman Disease preoperatively. Therefore, a combined diagnostic approach using both techniques, along with postoperative pathological examination, is recommended in clinical practice. CT and MRI provide a comprehensive assessment of the disease extent, determining whether the disease is confined to a single lymph node region (UCD) or involves multiple lymph node regions (MCD). Additionally, these imaging methods help identify any other lymphadenopathy or organ involvement. However, CT and MRI play a crucial role in the clinical diagnosis of CD. Enhanced CT is used to assess the degree of lymph node enhancement and to determine safe biopsy sites, while MRI helps further delineate the lesions and their anatomical relations. The typical histopathological subtype of UCD is the hyaline vascular type, which is closely related to its imaging features. On CT, UCD appears as an isolated, strongly enhancing, non-invasive soft tissue mass, typically 6-7 cm in diameter with clear margins. Smaller lesions usually display uniform density and enhancement, reflecting a rich vascular supply. For lesions larger than 5 cm, CT may show heterogeneous enhancement associated with areas of low density due to necrosis, fibrosis, or edema. Tortuous dilated feeding and draining vessels can often be seen near the lymph node masses, characteristic of CD. Arc-like, branching, or clustered calcifications may appear within the lesions. MRI holds significant value in CD for its ability to provide multiplanar anatomical depictions of masses and their relationship to adjacent structures, as well as highlighting the presence of feeding vessels. Lymph nodes or masses involved in CD most commonly appear as hypointense to isointense relative to skeletal muscle on T1-weighted images, and as isointense to hyperintense on T2-weighted images (17). At noncontrast MRI, these feeding vessels may appear as flow voids on T2-weighted images (12, 18). The presence of feeding vessels is sometimes considered a relatively specific feature of UCD, although this association can be variable (19).

The pathological subtypes of MCD mainly include the plasma cell type and mixed type. In comparison to UCD, MCD is characterized by an abundance of plasma cells mixed with hyaline vascular cells. MCD lacks distinctive features on CT, making it prone to misdiagnosis. It may present as systemic, multifocal lymphadenopathy and hepatosplenomegaly, easily confused with lymphoma. When distinguishing through imaging, UCD needs to be differentiated from highly vascular tumors such as thymoma, pheochromocytoma, and neurogenic tumors. Calcification and enhancement features aid in differentiation. Conversely, MCD needs to be distinguished from lymphoma, sarcoidosis, and lymph node tuberculosis. The threshold for distinguishing CD from lymphoma based on lymph node enhancement in enhanced CT is 92.5 Hounsfield units (HU), with values exceeding this threshold more likely representing CD (16).

CT, MRI, and pathological diagnosis are effective in differentiating the UCD in the presacral region reported in this case from other common presacral masses. The imaging features of Castleman disease on CT and MRI typically present as well-defined soft tissue masses with significant enhancement upon contrast administration, possibly with a central scar. Other presacral masses, such as chordomas, schwannomas, and teratomas, have distinct imaging characteristics. Chordomas generally exhibit aggressive bone destruction and soft tissue masses, with potential calcifications visible on CT. Schwannomas often appear as well-defined solid or cystic masses that enhance with contrast. Teratomas usually present as heterogeneous masses containing fat, soft tissue, and calcified elements.

The gold standard for diagnosing Castleman disease is pathological examination. Pathologically, Castleman disease shows proliferative lymphoid tissue with the characteristic “onion skin” appearance surrounding hyalinized vessels and central hyaline degeneration. Among other common presacral masses, chordomas originate from notochordal remnants, featuring physaliphorous cells and myxoid stroma under the microscope. Schwannomas are composed of Schwann cells, with spindle cells arranged in bundles pathologically. Teratomas contain various tissue components from all three germ layers, such as hair, teeth, and cartilage.

The pathological diagnosis of CD primarily relies on histological features, with different pathological subtypes distinguished through histological staining. In practical settings, pathologists integrate various staining methods, along with clinical presentations and imaging characteristics, for a comprehensive diagnosis of CD and its subtypes. Each staining method serves a specific purpose, aiding pathologists in more accurately identifying and classifying the disease.

Although we did not perform a preoperative biopsy, we recognize its importance. For certain specific locations of Castleman’s disease, locating the site for a preoperative biopsy and obtaining a specimen can be challenging. However, if a specimen can be obtained, a preoperative biopsy would be beneficial in differentiating other possible conditions such as lymphoma, sarcoidosis, and infectious diseases. It provides accurate pathological characteristics, which can help rule out other malignant or benign diseases (**Table 1**).

**Table 1:** Common tests used in the diagnosis of CD.

Staining Method	Purpose/Marker	Description
Hematoxylin and eosin (H&E)	Cellular structure and morphology	This is the most basic staining method, capable of revealing the cellular structure and morphology of tissues. In CD, features such as lymphoid follicle hyperplasia, vascular proliferation, and plasma cell infiltration can be observed.
Immunohistochemistry (IHC)	CD3, CD5, CD20, CD21	In hyaline vascular Castleman Disease, markers such as CD3, CD20, and CD21 could show diffuse expression. The follicular dendritic cell (FDC) network extends significantly both within and outside the germinal centers. Immunohistochemical staining, such as CD21 staining, reveals that FDCs strongly express the CD21 antigen. But these alone can’t make a complete diagnosis
	CD31 or Factor VIII	CD31, also known as PECAM-1, is a marker of endothelial cells primarily present at endothelial cell junctions. Factor VIII is a marker specific to vascular endothelial cells. In CD, CD31 or Factor VIII staining helps identify vascular proliferation features of the hyaline vascular type.
	Ki-67	Ki-67 is a nuclear protein expressed only during cell division. Therefore, Ki-67 labeling aids in evaluating cellular proliferative activity, crucial for assessing disease activity and potential malignant transformation. In CD, Ki-67 labeling can be used to assess the proliferation index of the lesion.
	IL-6	IL-6 is a pro-inflammatory cytokine involved in various physiological and pathological processes, including immune regulation, inflammation, and cancerogenesis. In CD, the overexpression of IL-6 is associated with the pathogenesis of HHV8-related multicentric CD. IL-6 detection helps identify inflammation-related cells, aiding in distinguishing disease subtypes (2, 20).
	HHV-8	Human herpesvirus 8 (HHV-8), also known as Kaposi’s sarcoma-associated herpesvirus (KSHV), is a member of the Herpesviridae family. Nearly all HIV-positive cases of multicentric CD and about 50% of HIV-negative cases are infected with HHV-8. However, studies have reported that in some cases of multicentric Castleman Disease without HIV co-infection, HHV-8 gene sequences can still be detected, suggesting that HHV-8 may also be a direct causative agent in multicentric Castleman Disease patients who are not co-infected with HIV (21, 22). In Castleman disease, HHV-8 staining assists in identifying viral infection, being valuable for the diagnosis of multicentric CD with vascular proliferation (23, 24).
	Congo red, CD10, CD30, EBER, IL-10, etc.	Congo red is a dye used for staining that selectively binds to β-folded proteins. In the diagnosis of Castleman disease, Congo red staining can help identify specific histological changes such as amyloidosis. In certain cases, additional markers such as CD10, CD30, EBER, IL-10, etc., can be used for specific identification (20, 25, 26).

Although the etiology of UCD remains unclear, its symptoms are generally mild and seldom life-threatening. UCD patients are usually asymptomatic or present with localized lymphadenopathy, with symptoms likely stemming from compression of adjacent structures by the swollen lymph nodes. Research by Li et al. (27) suggests that UCD may be a monoclonal proliferative disorder originating from lymph node interstitial cells. The diagnosis of HV-type UCD relies on pathological examination, including histological evaluation and immunohistochemistry (16). Imaging examinations such as CT or PET-CT can be utilized to delineate the extent of lesions and guide surgical interventions. For UCD, surgical excision aiming for maximal removal of the lesions is the preferred primary treatment modality. Following surgical resection of HV-type UCD, patients typically exhibit a favorable prognosis, with a survival rate exceeding 95% at 10 years among those who undergo resection (28). But long-term follow-up remains necessary after surgical excision of UCD, as there is a potential risk of recurrence.

## Conclusion

In this case study, we present a detailed description of the radiological features and pathological differential diagnosis process of a patient with presacral UCD. Radiological imaging, particularly CT and MRI, provided essential information regarding the location, size, and relationship with surrounding structures of the lesion, which was crucial for determining the surgical strategy. Histopathological examination, including HE staining and immunohistochemical analysis, further confirmed the diagnosis of UCD and revealed the histological characteristics of the lesion.

## Data Availability

The original contributions presented in the study are included in the article/supplementary materials. Further inquiries can be directed to the corresponding author.
